# Danggui Buxue Tang, a Traditional Chinese Herbal Formula, Potentiates Paclitaxel Efficacy in Non-Small-Cell Lung Cancer by Inducing Ferroptosis via the Nrf2/GPX4 Axis

**DOI:** 10.3390/ph19040607

**Published:** 2026-04-10

**Authors:** Guowei Gong, Tianpeng Yin, Zhenxia Zhang, Kumar Ganesan, Yuzhong Zheng

**Affiliations:** 1 Department of Bioengineering, Zunyi Medical University, Zhuhai Campus, Zhuhai 519041, China; ytp@zmu.edu.cn; 2 Guangdong Key Laboratory for Functional Substances in Medicinal Edible Resources and Healthcare Products, School of Life Sciences and Food Engineering, Hanshan Normal University, Chaozhou 521041, China; zzx8411@hstc.edu.cn (Z.Z.); zhengyuzhong@gmail.com (Y.Z.); 3 LKS Faculty of Medicine, School of Chinese Medicine, The University of Hong Kong, Hong Kong SAR, China; kumarg@hku.hk

**Keywords:** Danggui Buxue Tang, PTX, NSCLC, ferroptosis, Nrf2/GPX4 axis, oxidative stress

## Abstract

**Background/Objectives:** Non-small-cell lung cancer (NSCLC) involves oxidative stress and inflammation, driving chemoresistance. Paclitaxel (PTX), a first-line chemotherapy, is limited by these factors. Danggui Buxue Tang (DBT), a polyphenolic-rich traditional Chinese herbal formula, was investigated for its ability to potentiate PTX efficacy by inducing ferroptosis via the Nrf2/GPX4 axis. **Methods:** Effects of DBT + PTX on cell viability, lipid peroxidation, iron accumulation, and Nrf2/GPX4/SLC7A11 expression were evaluated in A549/HCC827 cells with/without ferrostatin-1 (Fer-1). Findings were validated in an A549 xenograft model. **Results:** DBT significantly enhanced PTX’s anti-tumor effects in vitro and in vivo, an effect reversed by Fer-1. Combination therapy increased ROS, MDA, and iron while suppressing GPX4/SLC7A11 and promoting Nrf2 nuclear translocation. DBT + PTX synergistically reduced tumor volume and proliferation markers (Ki67/PCNA). Crucially, DBT attenuated PTX-induced hepatotoxicity and nephrotoxicity. **Conclusions:** DBT potentiates PTX efficacy in NSCLC by disrupting the Nrf2/GPX4 axis to induce ferroptosis while mitigating chemotherapy-related toxicity, supporting its potential as an adjuvant strategy targeting oxidative stress pathways.

## 1. Introduction

Non-small-cell lung cancer (NSCLC) is a kind of lung cancer that accounts for around 85% of all lung cancer cases [1]. NSCLC is often diagnosed at an advanced stage, and the five-year survival rate is less than 20% [2]. Treatment options for NSCLC include chemotherapy, radiation therapy, and surgery. One of the most commonly used chemotherapy drugs for NSCLC is paclitaxel (PTX), which works by disrupting the microtubules in cancer cells and preventing them from dividing and growing [2]. However, the effectiveness of PTX can be limited by drug resistance and toxicity to healthy cells. Therefore, researchers have been investigating ways to enhance the therapeutic efficacy of PTXl and reduce its side effects [3]. One potential approach is to use traditional Chinese medicine (TCM) in combination with PTX. Several studies have investigated the potential of TCM in combination with chemotherapy drugs for the treatment of NSCLC. A meta-analysis of 21 randomized controlled trials involving 1565 patients with NSCLC found that TCM combined with chemotherapy drugs significantly improved overall response rate, performance status, and quality of life compared to chemotherapy alone [4,5].

Danggui Buxue Tang (DBT) is a TCM formula that has been used for centuries to treat various diseases, including cancer. It consists of Huangqi (*Astragali radix*, AR) and Danggui (*Angelicae sinensis radix*, ASR). With a history spanning centuries in TCM, DBT has been recognized for its ability to strengthen the immune system and promote overall well-being [6,7,8]. Recent research has delved into the pharmacological properties of DBT through both in vivo and in vitro studies [9,10,11]. These studies have revealed various effects of DBT administration in animal models, including increased red and white blood cell populations, estrogenic stimulation, bone regeneration promotion, immune response activation, and the induction of capillary and blood vessel formation [12,13,14,15,16]. Recent animal studies have provided additional evidence supporting the potential efficacy of DBT. For instance, in a study by Kong et al. [17], DBT administration in animal models increased red and white blood cell counts, indicating a potential hematopoietic effect. Similarly, muscle-derived stem cell transplantation after pretreatment with DBT in female Kunming mice improved the hematopoietic reconstitution [18]. Additionally, in a mouse model of aplastic anemia, DBT can restore hematopoietic function, repair hematopoietic failure, and alleviate immune-mediated death of hematopoietic stem cells [19]. Another study by Wang et al. [20] showed that DBT exhibited estrogenic effects and promoted bone regeneration in animal models of osteoporosis. The administration of DBT at the treatment sites of bone injury resulted in an increase in repair levels from 42.1% at week 4 to 71.2% at week 8, as demonstrated by micro-CT analysis [20]. The concentration of DBT that demonstrated the highest efficacy in the bone cell culture was 1000 μg/mL. While concurrently suppressing osteoclast activity, this dosage markedly raised the number of osteoblasts, intracellular alkaline phosphatase levels, and nodule counts [21]. Additionally, studies by Gong et al. demonstrated the ability of DBT to activate immune responses in animal models [22]. Indeed, the results of a randomized controlled clinical trial revealed fascinating findings. They demonstrated that administering DBT to patients with early-stage NSCLC after surgery effectively alleviated impairment and accelerated the recovery of immune function, ultimately enhancing the overall immunity of the patients [23]. These collective findings strongly suggest that DBT may have diverse physiological effects, as observed in in vitro studies, animal models, and clinical trials.

Recent investigations have explored the anti-cancer properties of DBT. Several studies have shown that DBT administration can enhance immune function and improve the effectiveness of chemotherapy in different types of cancers [17]. For instance, a study demonstrated that DBT enhanced the therapeutic efficacy of chemotherapeutic drugs in animal models of breast cancer [18]. Similar findings were reported in animal models of lung and liver cancer, respectively [19,20]. Studies have also suggested that DBT may enhance the therapeutic efficacy of chemotherapy drugs [24].

These studies highlight the potential of DBT as an adjunct therapy for cancer. Furthermore, recent investigations have demonstrated that DBT possesses anti-cancer properties and can improve immune function to enhance the effectiveness of chemotherapy in different types of cancer [25]. The primary active constituents of DBT, including astragaloside IV, calycosin, and ferulic acid, have been individually implicated in modulating oxidative stress and cell death pathways, suggesting they may serve as the mechanistic drivers of the formula’s chemosensitizing effects [26,27]. Studies have also suggested that DBT may enhance the therapeutic efficacy of chemotherapy drugs [24,28,29].

Ferroptosis differs from other forms of cell death like necrosis or apoptosis in that it is a sort of programmed cell death marked by the accumulation of iron-dependent lipid peroxides [30,31]. Since cancer cells frequently exhibit increased susceptibility to ferroptosis compared to normal cells, emerging evidence suggests that the induction of ferroptosis represents a promising and selective therapeutic strategy for cancer treatment [32]. The transcription factor nuclear factor erythroid 2-related factor 2 (Nrf2) is a master regulator of antioxidant responses, typically protecting normal cells from oxidative damage [33]. However, in the context of cancer, the role of Nrf2 is paradoxical. Constitutive activation of Nrf2, often due to mutations in its negative regulator KEAP1, can promote tumor progression and confer resistance to chemotherapeutic agents by upregulating a battery of cytoprotective genes, including heme oxygenase-1 (HO-1) and the ferroptosis-suppressing axis of SLC7A11 and glutathione peroxidase 4 (GPX4) [34,35,36,37,38,39,40,41]. This aberrant activation is associated with poor prognosis and chemoresistance in various cancers, including NSCLC [39,42]. Conversely, in some tumor contexts, Nrf2 and HO-1 may be downregulated, reflecting the complex, context-dependent nature of this pathway [42,43,44,45]. Despite these contextual variations, the Nrf2/GPX4 axis remains a critical protective mechanism against ferroptosis, as GPX4 plays a central role in eliminating lipid peroxides to prevent oxidative cell death [31,34,46,47,48]. Therefore, strategically disrupting this Nrf2-driven antioxidant defense represents a promising therapeutic strategy to sensitize cancer cells to chemotherapy-induced ferroptotic cell death.

In this study, we provide the first preclinical evidence that DBT—a traditional Chinese medicine formula standardized for its marker compounds ferulic acid and ononin—functions as a novel ferroptosis sensitizer. We demonstrate that DBT selectively disrupts the Nrf2/SLC7A11/GPX4 antioxidant defense axis in NSCLC cells, thereby potentiating the anti-tumor efficacy of PTX while concurrently mitigating its systemic toxicity. This work establishes a pharmacological rationale for repurposing DBT as an affordable, safe, and mechanism-based adjuvant to first-line NSCLC chemotherapy.

## 2. Results

### 2.1. Network Pharmacology Predication

**Figure 1:**
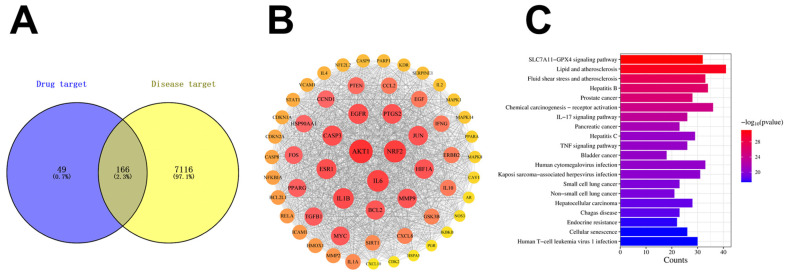
Network pharmacology prediction. (**A**) Venn diagram showing 166 overlapping targets between the 7282 NSCLC-associated targets and the 215 common targets for AR and ASR. (**B**) PPI network of the intersection targets. (**C**) KEGG pathway enrichment analysis of the identified targets.

To generate a hypothesis and identify potential mechanisms by which DBT might enhance PTX efficacy, we performed a network pharmacology analysis. This in silico approach predicted that 166 targets were common between the two herbs (AR and ASR) and NSCLC (Figure 1A). To further explore the relationships between these targets, a Protein–Protein Interaction (PPI) network was constructed, highlighting both direct and indirect regulatory interactions (Figure 1B). Following this, KEGG pathway enrichment analysis of these common targets highlighted several pathways, including those related to oxidative stress and ferroptosis (Figure 1C). This exploratory analysis led us to hypothesize that the anti-NSCLC effect of DBT + PTX might be mediated through the modulation of the Nrf2/GPX4 axis.

### 2.2. DBT Enhances the Therapeutic Functions of PTX in NSCLC, Which Is Associated with Ferroptosis and the Signal Transduction of the Nrf2/SLC7A11 Axis

**Figure 2:**
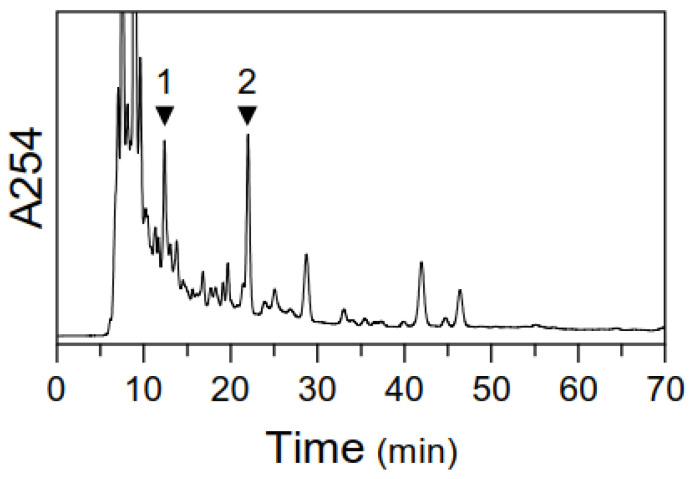
HPLC fingerprint of DBT for quality control. The chromatogram shows the chemical profile of the standardized DBT extract used in this study. Ferulic acid (peak 1, retention time ~13.5 min), a marker derived from *Angelicae sinensis radix* (ASR, Danggui), and ononin (peak 2, retention time ~23.5 min), a marker derived from *Astragali radix* (AR, Huangqi), were identified as the major active compounds. The presence of these peaks confirms the batch-to-batch consistency and chemical integrity of the DBT preparation, ensuring reproducibility of the pharmacological effects observed in both in vitro and in vivo experiments.

**Figure 3:**
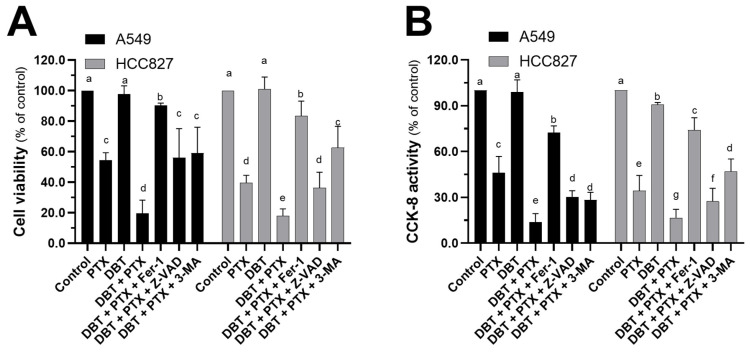
Fer-1 reverses DBT and PTX-induced cell death in the combination therapy. Cells were pretreated for 3 h with Fer-1 (1 μM), Z-VAD (10 nM), or 3-MA (1 μM), and then incubated for 2 days with different treatments (PTX at 1 μM, DBT at 0.5 mg/mL), or PTX and DBT (1 μM and 0.5 mg/mL) in combination. (**A**,**B**) The specific testing kit determined cell viability and/or CCK-8 activity. The data are presented as a percentage change from the control group and as Mean ± SEM, *n* = 3. Significantly different groups have different letters; otherwise they share the same letters.

Prior to conducting the biological assessment, we performed rigorous quality control on the DBT extract using HPLC. The chromatographic fingerprint, presented in Figure 2, confirmed the presence and consistency of two key bioactive markers: ferulic acid from ASR and ononin from AR [25]. This standardization step is critical for ensuring the reproducibility of our experimental results. The chemical validation of our DBT preparation provides a reliable foundation for the subsequent in vitro and in vivo pharmacological evaluations. DBT is a traditional herbal formula commonly used to enhance the therapeutic efficacy of chemotherapy agents in cancer patients. In our study, we conducted various in vitro assays to confirm the anti-NSCLC effects of the combination therapy involving DBT and PTX. Firstly, we performed cell proliferation, migration, and invasion assays to evaluate the effects of the DBT + PTX combination. Our results, as depicted in Figure 3A,B, showed that after 48 h of incubation, the combination therapy significantly suppressed cell proliferation and CCK-8 activity. Interestingly, the inhibitory effects of the combination therapy were greater than those observed with PTX alone, indicating that DBT enhanced the anti-NSCLC effects of PTX. Notably, at a concentration of 0.5 mg/mL, DBT alone did not induce cell death. To further investigate the underlying cell signaling pathways involved in the combination therapy, we utilized various pathway inhibitors. Our findings, as shown in Figure 3A,B, revealed that the ferroptosis inhibitor Fer-1 was able to reverse the inhibitory effects of the combination therapy. In contrast, the apoptosis inhibitor Z-VAD and the autophagy blocker 3-MA showed lower effectiveness compared to Fer-1, suggesting that the combination of DBT and PTX might promote NSCLC ferroptosis to enhance its anti-cancer functions.

**Figure 4:**
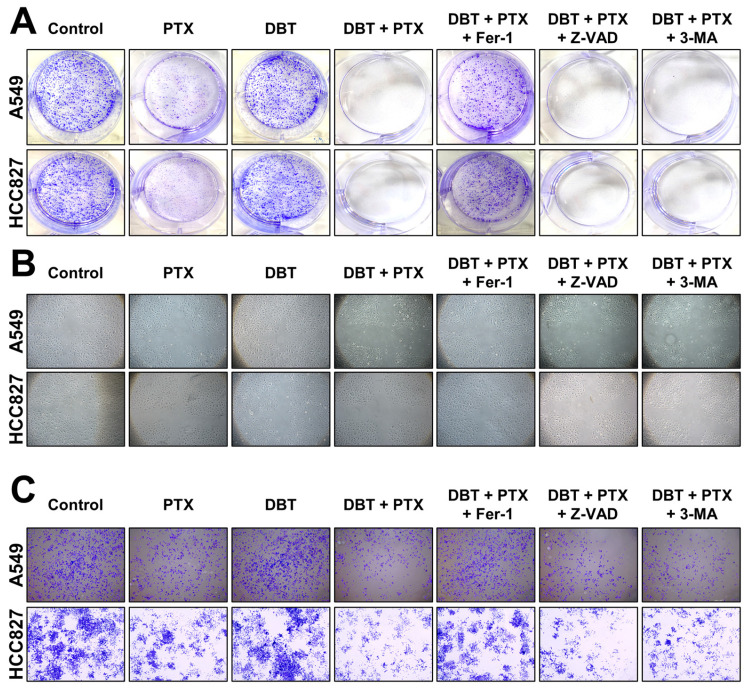
Fer-1 reverses the combination therapy-induced anti-colony formation and anti-cell migration functions. Cells were pretreated for 3 h with Fer-1 (1 μM), Z-VAD (10 nM), or 3-MA (1 μM), and then incubated for 2 days with different treatments (PTX at 1 μM, DBT at 0.5 mg/mL), or PTX and DBT (1 μM and 0.5 mg/mL) in combination. (**A**) After 48 h of drug treatment, cells were seeded in a 6-well plate and switched to fresh medium for another 7 days. The colony formation was covered with crystal violet. (**B**) Cells from culture were seeded into a 12-well plate. Cells were artificially wounded by scratching the cell monolayer at the bottom of wells, and wound images were captured after drug treatment for 2 days. (**C**) Cells were placed in the top layer of each well of a transwell plate. After 48 h of treatment with various medications, the bottom layer was fixed and stained.

To assess cell migration activities following 48 h of DBT + PTX combination therapy incubation, we conducted colony formation, wound healing, and transwell assays (Figure 4). Our results showed that, compared to the blank control group, PTX alone was able to reduce NSCLC colony formation, wound healing, and migration (Figure 4A–C). However, DBT + PTX exhibited superior anti-colony formation and anti-migration functions compared to PTX alone, indicating that the combination therapy had a more effective role in treating cancer in vitro. Furthermore, Fer-1 was able to reverse the decline in colony formation and cell migration activities triggered by the combination therapy (Figure 4A–C). These findings suggest that the anti-NSCLC effects of the combination therapy primarily involve ferroptosis. Overall, our study provides valuable insights into the potential application of DBT as an adjunct to chemotherapy agents for the treatment of NSCLC.

**Figure 5:**
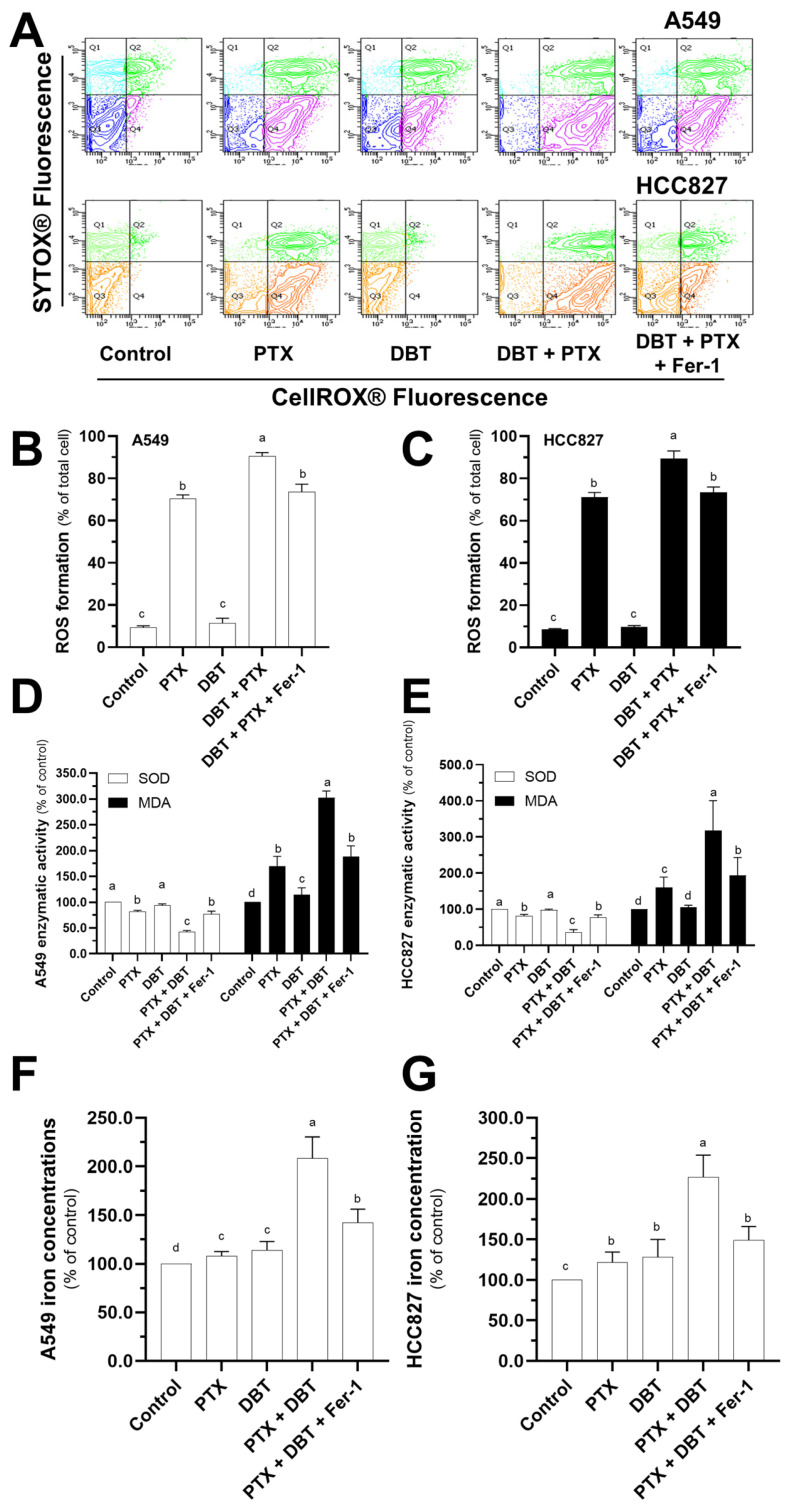
Fer-1 reverses the ROS formation and ferroptosis caused by combination therapy in NSCLCs. Cells were pretreated for 3 h with Fer-1 (1 μM) before being incubated for 2 days with various treatments (PTX at 1 μM, DBT at 0.5 mg/mL, or PTX and DBT (1 μM and 0.5 mg/mL) in combination. (**A**) Flow cytometer was used to detect ROS formation using CellROX^®^ detection kit (Thermo Fisher Scientific, Grand Island, NY, USA). (**B**,**C**) The quantification analyses were carried out using the FlowJo v10.6 software. (**D**–**G**) The specific kits used to measure SOD and MDA enzymatic activity and cellular iron concentration in NSCLCs were used as directed by the manufacturer. Significantly different groups have different letters; otherwise they share the same letters.

We measured the production of reactive oxygen species (ROS) in various drug treatment groups using a flow cytometer (Figure 5A). Interestingly, the combination therapy group (DBT + PTX) induced higher ROS production after 48 h of treatment compared to the PTX medication group. This was evident when comparing the ROS production in the PTX group to the control group (Figure 5A–C). The increase in ROS production caused by the combination therapy could be reduced by pre-treating the cells with Fer-1 (Figure 5A–C). To gain further insights into the anti-cancer mechanism of the combination therapy, we investigated several indicators related to ferroptosis (Figure 5D–G). In both A549 and HCC827 cells, the co-treatment of PTX and DBT significantly increased the cellular accumulation of iron and malondialdehyde (MDA) (Figure 5D–G). Additionally, after a 48 h incubation period, the combination therapy appeared to decrease the superoxide dismutase (SOD) level. However, these effects could be abolished by Fer-1 (Figure 5D–G). These findings suggest that the combination therapy induces ROS production and affects indicators associated with ferroptosis. This information contributes to our understanding of the anti-cancer mechanism of the combination therapy involving DBT and PTX.

**Figure 6:**
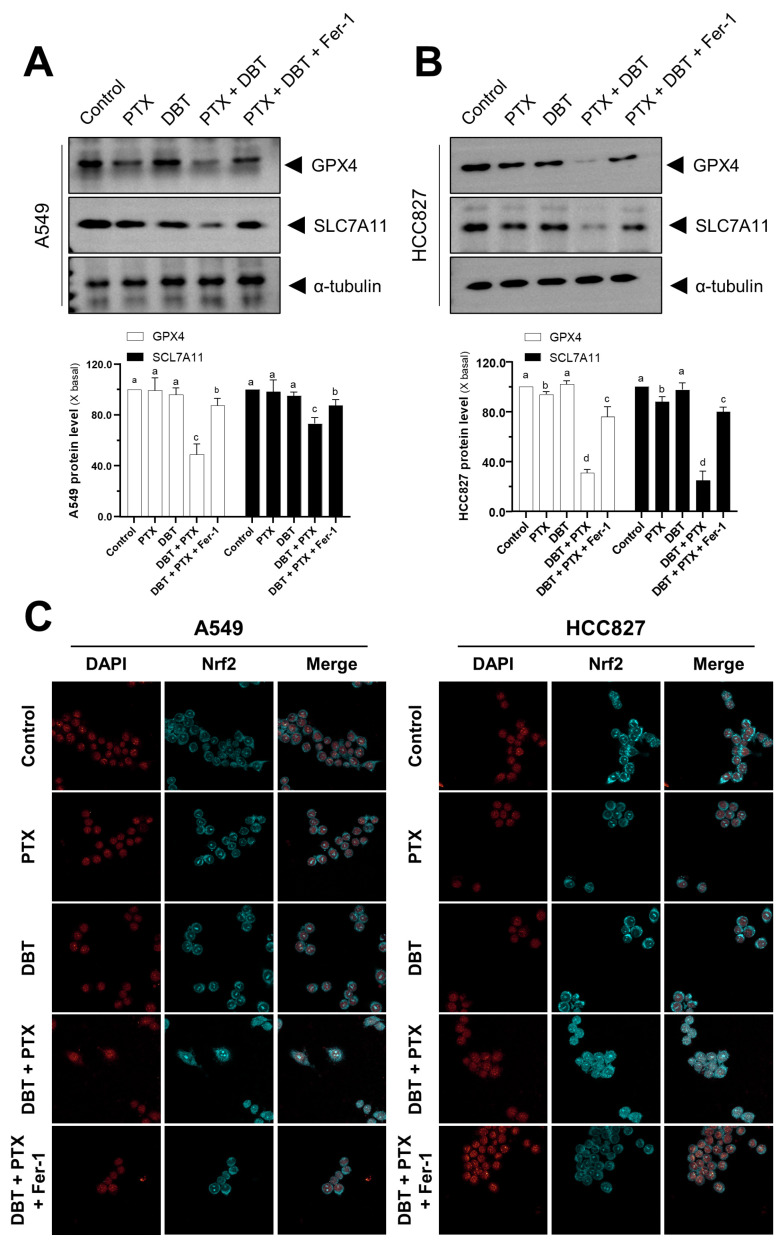
Combination therapy with PTX and DBT alters Nrf2/GPX4/SLC7A11 protein expression levels in NSCLCs. Cells were pretreated with Fer-1 (1 μM) for 3 h before being incubated for 2 days with various treatments (PTX at 1 μM, DBT at 0.5 mg/mL, or PTX and DBT (1 μM and 0.5 mg/mL in combination). (**A**,**B**) Total protein was extracted and immunoblotted with GPX4 and SLC7A11 antibodies. The fold change compared to the blank control group and the Mean ± SEM, *n* = 3, are used to express the data. Significantly different groups have different letters; otherwise they share the same letters. (**C**) The presence of Nrf2 (blue) and nuclei (red) was revealed by immunofluorescence.

We also examined the protein expression levels in cultured cells to investigate whether the combination therapy induces ferroptosis. Western blot analysis showed that in both A549 and HCC827 cells, the levels of GPX4 and SLC7A11 were reduced by the combination therapy (Figure 6A,B). However, this decline in GPX4/SLC7A11 levels could be reversed by pre-treating the cells with Fer-1 (Figure 6A,B). Furthermore, we assessed the Nrf2 translocation activity in response to different drug treatments (Figure 6C). We observed that the combination therapy of DBT + PTX caused Nrf2 to translocate from the cytosol to the nucleus. However, this effect could be reversed by Fer-1 treatment (Figure 6C). Taken together, these findings indicate that the DBT + PTX combination disrupts the Nrf2/SLC7A11/GPX4 antioxidant defense axis, leading to ferroptosis in NSCLC cells.

### 2.3. DBT Improves PTX Pharmacological Activities in Xenograft Mice by Promoting Ferroptosis

**Figure 7:**
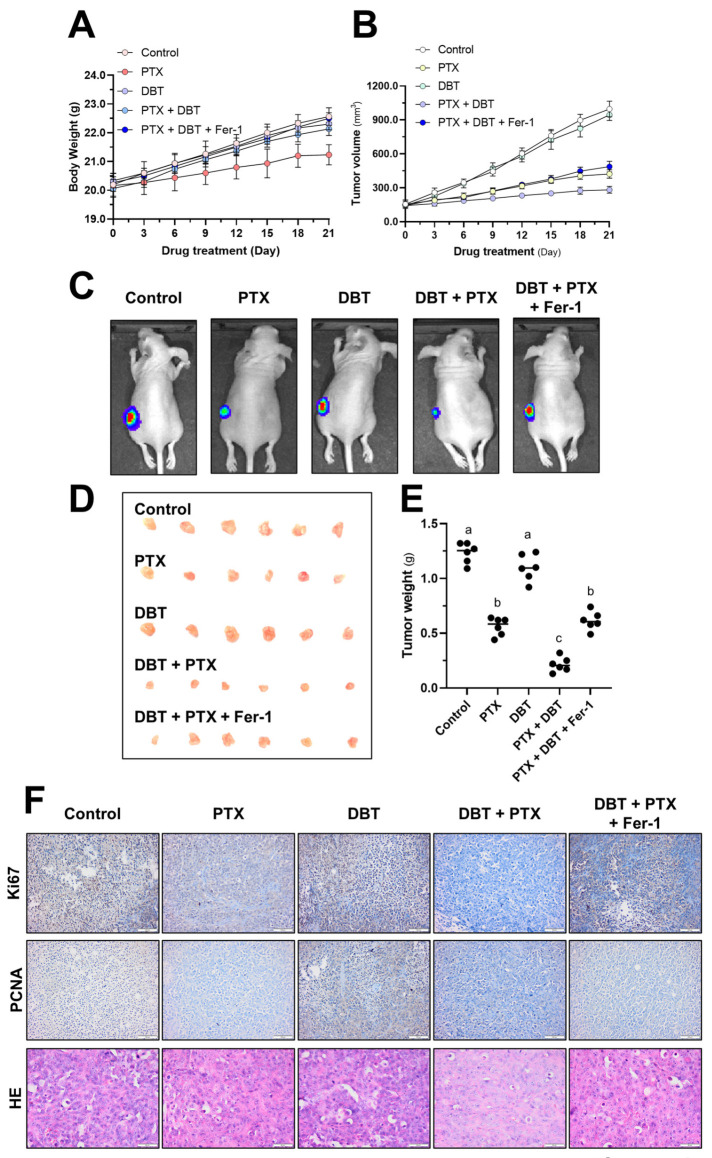
The anti-NSCLC functions of the combination therapy in vivo. The control group received Milli-Q water; the PTX group received PTX (2 mg/kg); the DBT group received DBT herbal formula (10 mg/kg); and PTX was combined with the DBT group (2 mg/kg and 10 mg/kg). PTX + DBT + Fer-1 received 2 mg/kg + 10 mg/kg + 2 mg/kg. (**A**,**B**) During the drug treatment, body weight and tumor volume were measured every three days. (**C**) Bioluminescence imaging of a nude mouse xenograft tumor after 21 days of therapy. (**D**) Tumor tissue was isolated from various groups. (**E**) The weight of tumor tissue was calculated. The data is expressed using the fold change compared to the blank control group and the Mean ± SEM, *n* = 6. Significantly different groups have different letters; otherwise they share the same letters. (**F**) Immunohistochemistry was used to examine Ki67 and PCNA expression, and the HE staining of tumor tissue is shown here.

In our study, we developed A549 xenograft mice to investigate whether DBT enhances the therapeutic effects of PTX by inducing ferroptosis in vivo (Figure 7). Throughout the treatment duration, we recorded the body weight of the mice (Figure 7A). The mice treated with PTX showed a significant decrease in body weight, while no weight loss was observed in the other groups (Figure 7A). Importantly, compared to chemotherapy alone, the combination therapy of DBT and PTX significantly reduced tumor volume (Figure 7B). However, in the PTX + DBT + Fer-1 group, the tumor volume measured was larger compared to the PTX + DBT group (Figure 7B). We used an in vivo imaging system to determine the size of the tumors in the xenograft mice (Figure 7C). The fluorescent intensity analysis showed that PTX significantly suppressed tumor growth compared to the control group (Figure 7C). The PTX + DBT treatment further reduced tumor size, but this therapeutic effect could be reversed by Fer-1 (Figure 7C–E).

To assess the proliferation of tumor cells, we performed immunohistochemical (IHC) analysis of the tumor tissue, specifically staining for Ki67 and PCNA, which are markers associated with proliferation (Figure 7F). We found that PTX significantly decreased the levels of Ki67 and PCNA compared to the control and DBT groups (Figure 7F). Importantly, in the combination therapy group, the levels of Ki67 and PCNA were significantly lower compared to the PTX-alone group (Figure 7F). Notably, Fer-1 could compensate for the downregulation of Ki67 and PCNA levels in the combination therapy group (Figure 7F). Furthermore, HE staining of the tumor tissue revealed that the combination therapy group had a higher concentration of dead cells compared to the untreated or single-medication groups (Figure 7F). Additionally, the anti-cancer effects induced by PTX + DBT could be restored by Fer-1 (Figure 7F). Overall, our in vivo results demonstrate that the combination therapy of DBT and PTX effectively reduces tumor volume, inhibits proliferation and induces cell death. These effects can be reversed by Fer-1, indicating the involvement of ferroptosis in the anti-cancer mechanism of the PTX + DBT combination therapy.

### 2.4. Evidence of Ferroptosis-Related Mechanisms in the Anti-Cancer Effects of DBT + PTX Combination Therapy: Insights from In Vivo Analysis

**Figure 8:**
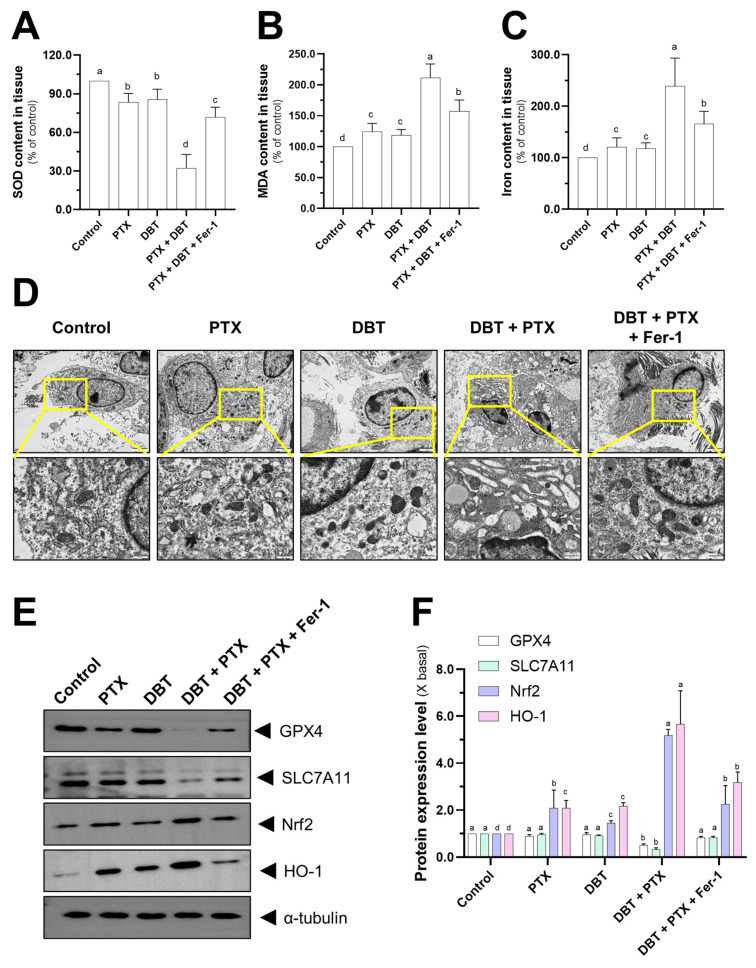
Combination therapy can cause ferroptosis and affect the Nrf2/GPX4/SLC7A11 axis. (**A**–**C**) In the homogenized tumor tissues, the kit was used to measure SOD, MDA, and cellular iron concentrations. (**D**) TEM revealed that combination therapy reduced mitochondria and cristae in tumor tissues. (**E**,**F**) The target gene expression activities in tumor tissue were determined using a Western blot. The target protein’s quantification was calculated using a densitometer. The values are given as the fold change (X basal), in Mean ± SEM, with *n* = 6. Significantly different groups have different letters; otherwise they share the same letters.

In order to gain further understanding of the anti-cancer mechanism of the combination therapy, we investigated various indicators related to ferroptosis (Figure 8). The combination therapy significantly decreased the levels of superoxide dismutase (SOD), while increasing the levels of malondialdehyde (MDA) and iron in the tumor tissue (Figure 8A–C). Fer-1 treatment was able to reverse these effects (Figure 8A–C). We also used TEM to monitor morphological changes in the mitochondria following different drug treatments (Figure 8D). We found that the DBT + PTX combination therapy may reduce mitochondrial volume while increasing mitochondrial membrane density (Figure 8D). Western blot analysis revealed that the combination therapy group had significantly lower expression levels of the ferroptosis-related proteins SLC7A11 and GPX4 (Figure 8E,F). Interestingly, the expression levels of Nrf2 and HO-1 were increased after combination therapy treatment compared to the control group (Figure 8E,F). Fer-1 treatment reversed the expression levels of GPX4, SLC7A11, Nrf2, and HO-1. These findings indicate that Fer-1 reduces the ferroptosis induced by the PTX + DBT combination therapy in A549 xenograft mice. Furthermore, the in vivo data is consistent with the results obtained from cell culture experiments (Figure 8E,F).

### 2.5. Combination Therapy Demonstrates Reduced Toxicity In Vivo: Evidence from Biochemical and Histological Analysis

**Figure 9:**
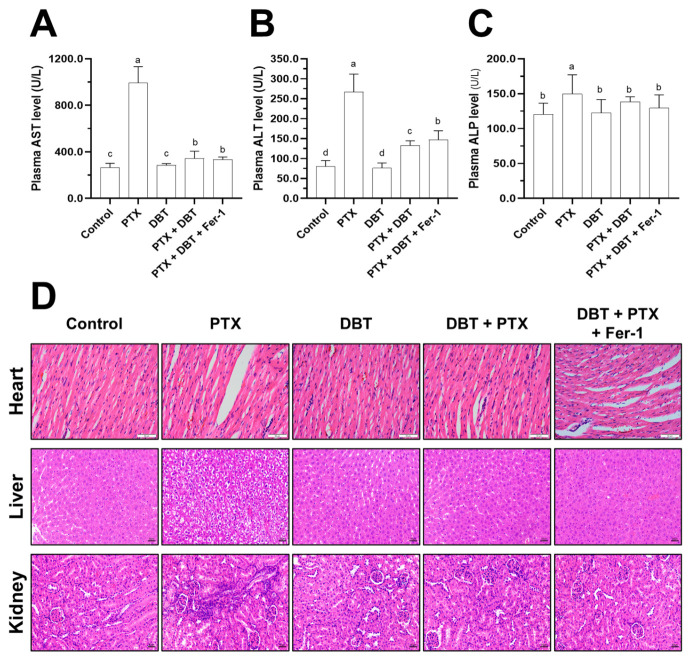
Combination therapy shows biosafety in the xenograft mice. (**A**–**C**) Detection of AST, ALT and ALP in nude mice. The values are given in Mean ± SEM, with *n* = 6. Significantly different groups have different letters; otherwise they share the same letters. (**D**) HE staining of heart, liver and kidney in the xenograft mice obtained from different groups.

In order to assess the possible toxicity of the combination medications in vivo, we measured the levels of AST, ALT, and ALP, which are established serum markers of liver function. The results showed that PTX alone significantly increased the levels of these markers, indicating hepatotoxicity. Remarkably, the combination therapy (DBT + PTX) was able to normalize these elevated concentrations after 21 days of treatment (Figure 9A–C), suggesting a protective effect against PTX-induced liver damage. Additionally, we examined various organs obtained from the different treatment groups using H&E staining (Figure 9D). No apparent tissue lesions were observed in the histological samples from the combination therapy experimental groups (Figure 9D). Additionally, there was no significant indication of cardiac fibrosis, liver inflammation, or glomerular or tubular changes in the kidneys (Figure 9D). In contrast, mice treated with PTX exhibited notable morphological alterations in the livers and kidneys compared to the other groups (Figure 9D). These results suggest that ferroptosis plays a role in the tumor growth inhibition mediated by PTX + DBT through the Nrf2/GPX4 axis, both in vitro and in vivo. Furthermore, the combination therapy did not appear to cause significant toxicity or organ damage, as evidenced by normal levels of AST, ALT, and ALP, along with the absence of visible tissue lesions or histopathological changes in the organs assessed.

## 3. Discussion

PTX has been acknowledged as a chemotherapeutic agent with significant clinical effectiveness against various cancer types and is now considered the first-line treatment for NSCLC [2,49]. However, prolonged use and optimal doses of this drug may result in severe side effects, such as neutropenia, neuropathy, and cancer cell resistance [5]. Currently, there is a growing interest in discovering new therapeutic agents with synergistic effects when combined with PTX. Increasing evidence suggests that combination therapies are more effective than monotherapy in treating cancer [50,51].

Combining multiple drugs not only enhances the effectiveness of each individual drug and allows for lower doses of a single agent, but also reduces the likelihood of drug resistance [30,52]. There is evidence suggesting that the combination of plant-derived active compounds and chemotherapeutic drugs can provide significant therapeutic benefits. Plant-derived compounds generally have improved efficiency and reduced toxicity, and can enhance immunity by regulating various signaling pathways.

AR and ASR, two herbs included in DBT, have a long-standing history of use in TCM and are well-known for their wide range of pharmacological effects in Asian countries. These herbs demonstrate a broad spectrum of pharmacological effects, such as anti-inflammatory, antioxidant, proangiogenic, and anticarcinogenic activities [6,12,13,14]. AR is a significant medicinal plant that exhibits a wide range of pharmacological activities. These include immunomodulatory effects, protection of the heart and liver, antioxidant properties, and potential anti-cancer properties [53,54,55]. AR has demonstrated beneficial effects on different types of cancer, such as hepatocarcinogenesis, colon, lung, and gastric carcinoma [55,56,57,58]. It is recognized as an antineoplastic agent that can improve treatment efficacy and minimize toxicity during radiotherapy or chemotherapy. Furthermore, a clinical trial has suggested that AR may enhance the effectiveness of PTX-based chemotherapy in patients with NSCLC [56]. The significant role and synergistic mechanisms of AR in combination with cisplatin in the treatment of laryngeal squamous cell carcinoma have been well documented [59]. This suggests that the use of AR in combination with cisplatin can lead to enhanced therapeutic effects against laryngeal squamous cell carcinoma.

In terms of tumor growth inhibition, the combination therapy of PTX and DBT significantly reduced tumor volume compared to chemotherapy alone. This effect was further supported by in vivo imaging, which showed a significant suppression of tumor growth in the PTX-treated group. Importantly, the therapeutic effects of the combination therapy were reversed when Fer-1, a ferroptosis inhibitor, was administered. These results indicate that the induction of ferroptosis plays a crucial role in the anti-cancer effects of the PTX + DBT combination therapy. This study is consistent with our previous studies showing that the combination of a TCM formula along with PTX significantly reduced tumor volume and size [2].

Further investigations into the mechanisms underlying the induction of ferroptosis revealed several important findings. The PTX + DBT combination therapy led to decreased levels of SOD, an antioxidant enzyme, while increasing levels of MDA and iron in the tumor tissue. These changes indicate an imbalance in redox homeostasis and the accumulation of lipid peroxides, which are key features of ferroptosis. These findings align with our previous reports demonstrating that TCM formulas can enhance PTX efficacy by promoting ferroptotic cell death [24,30,60]. Additionally, TEM analysis showed that the combination therapy caused morphological alterations in the mitochondria, including reduced mitochondrial volume and increased mitochondrial membrane density. These changes in mitochondrial morphology are consistent with previous studies suggesting a link between mitochondrial dysfunction and ferroptosis [61,62,63]. At the molecular level, the combination therapy resulted in decreased expression of the ferroptosis-related proteins SLC7A11 and GPX4, which are crucial for maintaining cellular redox balance and preventing lipid peroxidation. This decrease in SLC7A11 and GPX4 expression indicates a disruption of the cellular antioxidant defense system, promoting ferroptosis. Interestingly, the PTX + DBT combination therapy also led to increased expression of Nrf2 and HO-1, which are transcription factors involved in cellular stress response and antioxidant defense. This upregulation of Nrf2 and HO-1 may represent a compensatory mechanism to counteract the ferroptotic process induced by the combination therapy of PTX + DBT. Importantly, the administration of Fer-1 reversed the expression levels of GPX4, SLC7A11, Nrf2, and HO-1, supporting the notion that Fer-1 can attenuate the ferroptotic effects of the combination therapy. In terms of safety, the combination therapy did not induce significant toxicity or organ damage.

A key finding of this study is the apparent paradox of Nrf2 nuclear translocation alongside the downregulation of its canonical targets, GPX4 and SLC7A11. While Nrf2 is a well-established transcriptional activator of SLC7A11 and GPX4 [26,37,64], our data suggest a more complex scenario. We propose that the DBT + PTX combination induces a state of overwhelming oxidative stress and lipid peroxidation. This stress initially triggers a compensatory Nrf2 nuclear translocation, an attempt by the cell to mount a protective response. However, the combination therapy likely disrupts the integrity of the Nrf2 transcriptional machinery or promotes the degradation of GPX4 and SLC7A11 proteins, overriding this protective feedback loop. Consequently, the increase in Nrf2 activity becomes a ‘futile’ response, uncoupled from its downstream antioxidant effectors. The observation that Fer-1, which blocks ferroptosis, also reverses Nrf2 nuclear translocation supports the idea that Nrf2 activation is a consequence, rather than the primary initiator, of the oxidative stress and ferroptosis induced by the DBT + PTX combination. This underscores that the core mechanism is the effective disruption of the antioxidant defense, leading to lethal lipid peroxidation.

While our data firmly establish the involvement of the Nrf2/SLC7A11/GPX4 axis in mediating the anti-cancer effects of DBT + PTX, we acknowledge that ferroptosis is regulated by a complex and multifaceted network. Beyond the canonical SLC7A11/GPX4 pathway, other independent ferroptosis defense systems exist, including ferroptosis suppressor protein 1 (FSP1), GTP cyclohydrolase 1 (GCH1), and the CoQ10 biosynthesis pathway [26,37,40,41]. These parallel systems can compensate for GPX4 loss in certain cellular contexts and may contribute to resistance mechanisms. Future studies are warranted to investigate whether these GPX4-independent ferroptosis pathways are also modulated by the DBT + PTX combination and whether they play a role in the observed anti-tumor efficacy. The levels of AST, ALT, and ALP, which are indicators of liver and kidney function, were within the normal range after treatment [65,66]. Furthermore, histological examination of various organs did not reveal any visible tissue lesions or histopathological changes. This suggests that the combination therapy of PTX + DBT has an acceptable safety profile.

These findings are consistent with earlier publications that have reported the involvement of ferroptosis in cancer therapy [40,43,67,68,69]. Ferroptosis has emerged as a promising target for cancer treatment due to its unique mode of cell death and its potential to overcome resistance to conventional therapies [26,32,70,71]. The induction of ferroptosis by the PTX + DBT combination therapy, as demonstrated in this study, provides a rationale for further exploring this therapeutic approach in NSCLC and potentially other cancer types. It is worth noting that calycosin, formononetin, and ferulic acid have been reported to exhibit anti-cancer effects in many plants [27,72]. However, further comprehensive pharmacological and toxicological investigations are required to fully explore the potential of these compounds as anti-cancer agents. Our laboratory is currently studying which of these possibilities are the major factors contributing to the anti-cancer role of DBT.

DBT decoction contains a complex mixture of bioactive phytochemicals, and its pharmacological efficacy is likely driven by the synergistic action of multiple active principles rather than a single compound. The key constituents identified in our standardized extract include astragaloside IV, calycosin, ononin, and formononetin from AR, as well as Z-ligustilide and ferulic acid from ASR [6].

Ferulic acid, a phenolic compound derived from ASR, is well-documented for its potent antioxidant and anti-inflammatory properties. However, recent evidence suggests it can also modulate redox balance in cancer cells, potentially sensitizing them to oxidative stress-induced death such as ferroptosis [27,72]. Calycosin and formononetin, major isoflavonoids from AR, have demonstrated direct anti-cancer activities by inducing apoptosis and inhibiting metastasis in lung and breast cancer models [72]. Notably, calycosin has been shown to orchestrate the hematopoietic and estrogenic functions of DBT, suggesting its role as a key orchestrator within the formula [18,19].

Furthermore, astragaloside IV, a saponin from AR, has recently been implicated in the regulation of ferroptosis via the Nrf2/SLC7A11/GPX4 axis in models of lung injury [26]. This aligns directly with our findings, where DBT + PTX combination therapy disrupted this same axis to induce ferroptosis. It is plausible that astragaloside IV, in concert with calycosin and ferulic acid, contributes to the synergistic enhancement of PTX efficacy observed in our study.

While our data clearly demonstrate the enhanced anti-cancer effect of the DBT + PTX combination, it is important to note that this efficacy likely results from the cumulative and synergistic interactions of these active ingredients. Future studies in our laboratory are focused on using chemical knock-out methodologies and combinatorial pharmacological approaches to dissect the specific contribution of each of these compounds to the ferroptosis-inducing and chemosensitizing effects of DBT. Determining whether a single agent or a specific combination of these active principles is the primary driver of the observed effects will be crucial for the future development of DBT as a standardized adjuvant therapy.

The DBT extract used in this study was standardized based on the presence of its two key bioactive markers, ferulic acid and ononin, to ensure batch-to-batch consistency. However, we acknowledge that this is a simplification of a complex chemical matrix. A more comprehensive chemical fingerprint, quantifying other known active components such as astragaloside IV, calycosin, and formononetin, would be beneficial for future studies to fully correlate specific chemical profiles with biological activity.

This differential effect highlights a critical therapeutic advantage: DBT selectively sensitizes cancer cells to ferroptosis while concurrently protecting healthy organs from the toxic side effects of chemotherapy. From a translational perspective, the favorable safety profile of DBT—demonstrated by normalized liver and kidney function markers and the absence of histopathological damage—positions this combination regimen as a viable candidate for clinical evaluation. Given that DBT is already used clinically in China for postoperative immune recovery in NSCLC patients [23], the repurposing of this well-tolerated herbal formula as a ferroptosis-inducing adjuvant to PTX chemotherapy represents a low-risk, high-reward strategy. Future phase I/II clinical trials should focus on validating the optimal DBT dosage required to achieve ferroptosis-specific biomarkers in patient tumor tissues.

### Limitations and Future Perspectives

Although our study was limited to a single-dose experiment in both the in vitro and in vivo investigations, it is important to acknowledge the potential benefits of exploring a broader range of concentrations in future studies. Including multiple test concentrations for each treatment would provide a more comprehensive understanding of the potential dose-dependent effects and the claimed synergistic/enhanced effect and pharmacodynamics. This would allow for a more robust scientific insight into the treatment’s mechanisms of action and therapeutic efficacy. Future research should consider conducting dose–response studies to determine the optimal concentration range for achieving the desired effects. Additionally, investigating the potential synergistic interactions between different concentrations or combinations of treatments could provide valuable insights into the treatment’s combinatorial therapeutic potential.

Several additional limitations should be acknowledged. First, while our in vitro results demonstrate that the DBT + PTX combination inhibits NSCLC cell proliferation more effectively than PTX alone, formal synergy analysis using models such as Bliss independence or Chou-Talalay combination index was not performed in the current study. Such analysis would be valuable to quantitatively distinguish between additive and synergistic effects and to determine the optimal dose ratio for combination therapy. Future studies should incorporate these approaches to provide a more rigorous pharmacological characterization of the interaction between DBT and PTX.

Second, the conclusion that ferroptosis is the primary mechanism underlying the enhanced anti-cancer effect of DBT + PTX was primarily based on the reversal of cell death by ferrostatin-1 (Fer-1), a well-established ferroptosis inhibitor. However, Fer-1 functions as a lipophilic antioxidant that traps lipid peroxyl radicals, and its effects, while indicative of ferroptosis, are not entirely specific to this cell death pathway. To definitively establish ferroptosis as the central mechanism, future studies should employ complementary approaches, including the use of structurally distinct ferroptosis inhibitors such as liproxstatin-1, genetic knockdown of key ferroptosis effectors (e.g., GPX4 or SLC7A11), and direct measurement of additional ferroptosis-specific markers such as ACSL4 activation and phospholipid hydroperoxide accumulation. These investigations would provide more conclusive evidence for the mechanistic role of ferroptosis in the observed therapeutic synergy. These future studies will contribute to the advancement of our understanding and the optimization of this treatment approach.

## 4. Materials and Methods

### 4.1. Network Pharmacology Analysis

The AR and ASR target proteins were predicted using the Chinese Traditional Medicine System Pharmacological Database (https://www.tcmsp-e.com/index/, accessed on 2 January 2021) and the SuperPred database (https://prediction.charite.de/, accessed on 2 January 2021). Disease-related targets were discovered using publicly available databases: GeneCards (https://www.genecards.org/, accessed on 2 January 2021) and OMIM (https://www.omim.org/, accessed on 2 January 2021). The keywords used in the search were “non-small cell lung cancer” and “NSCLC.” The overlapping targets of raw materials and NSCLC were entered into the STRING 11.5 database (https://cn.string-db.org/, accessed on 2 January 2021) to create a Protein–Protein Interaction (PPI) network, which was then displayed with Cytoscape software (version 3.7.2). Pathway enrichment analysis was conducted using the Metascape platform, focusing on the Kyoto Encyclopedia of Genes and Genomes (KEGG) pathways.

### 4.2. HPLC Condition

The analysis was performed using an Agilent 1200 series system (Agilent, Waldbronn, Germany), equipped with a thermo-stated column compartment, an auto-sampler, a binary pump, and a degasser. Chromatographic separation was carried out on an Agilent Eclipse Plus C18 column (4.6 × 250 mm, 5 µm) at room temperature. The mobile phase consisted of acetonitrile (Solvent A) and 0.01% formic acid (Solvent B), with a flow rate of 1.0 mL/min. A linear gradient elution was applied, ranging from 15% to 80% Solvent A over 0 to 70 min. Samples were filtered through a Millipore syringe filter unit with a 0.45 µm filter before analysis. For HPLC, 10 µL of each sample was injected, and detection was performed at a wavelength of 254 nm.

### 4.3. Raw Materials and DBT Preparation

The morphological identification of the herbs, AR and ASR, was based on the China Pharmacopeia (2025). The voucher specimens have been deposited at Zunyi Medical University in Zhuhai, China, with voucher numbers #YP-201-07-01 for ASR and #YP-201-07-02 for AR. Exact amounts of AR and ASR were weighed in accordance with a 5:1 ratio and thoroughly mixed before being used to prepare DBT herbal extracts. The preparation followed a traditional decoction method: the mixture was boiled in eight volumes of water (*v*/*w*) for two hours, and the extraction process was repeated twice to ensure maximal extraction of bioactive constituents. After lyophilization, the extracts were dried and kept at −80 °C. To prepare it for biological testing and HPLC analysis, the sample was redissolved in water. The chemical profile of the extract was characterized by HPLC, with ferulic acid and ononin selected as marker compounds to ensure batch-to-batch consistency, as shown in Figure 2.

### 4.4. Cell Culture

The A549 and HCC827 cells were provided by the American Type Culture Collection (ATCC) based in Manassas, VA. These cell lines were cultured in Dulbecco’s modified Eagle’s medium supplemented with 10% fetal bovine serum, 100 g/L streptomycin, and 100 IU/mL penicillin. The cells were incubated at 37 °C in a CO_2_ incubator with 5% CO_2_ supply.

### 4.5. MTT and CCK-8 Assays

Cell viability was assessed using MTT and/or CCK-8 testing kits. All chemicals and reagents were provided by Sigma-Aldrich (St. Louis, MO, USA). The cells were plated and grown in 96-well plates. PTX and DBT were used at working concentrations of 1 μM and 0.5 mg/mL, respectively. The dosage of DBT and PTX was determined based on previous publications [25,64,73]. Ferrostatin-1 (Fer-1), Z-VAD-FMK (Z-VAD), and 3-methyladenine (3-MA) were used at working concentrations of 1 μM, 10 nM, and 1 μM, respectively. After 48 h of treatment, the MTT solution (0.5 mg/mL) was added to the cell cultures. After a two-hour incubation, the purple crystals were dissolved in dimethyl sulfoxide (DMSO), and the absorbance at 570 nm was measured for calibration. For the CCK-8 assays, absorbance was measured at 450 nm using a Multiskan GO plate reader (Thermo Fisher Scientific, Grand Island, NY, USA). Cell viability was calculated by determining the percentage of absorbance relative to the control group, which was set to 100%.

### 4.6. Colony Formation Assay for Analyzing Cell Proliferation Efficacy

In a 6-well plate, 1000 cells were cultured for 48 h in the presence of a different medicine. Afterward, the culture medium was replaced with fresh medium and the cells were further incubated for an additional 7 days. To fix the cells, they were treated with methanol for 15 min at room temperature. Subsequently, the cells were stained with crystal violet (Sigma-Aldrich) for 10 min.

### 4.7. Wound Healing Assay

To evaluate the motility of cells treated with different medications, a wound healing assay was performed. Cells were cultured in 12-well plates and allowed to grow to 90–100% confluence. A pipette tip was then used to create a wound by scraping through the monolayer. The detached cells were removed by washing with PBS, and serum-free medium containing varying concentrations of the medications was added. After 48 h of incubation, images were captured using a microscope with a 10× objective (Leica, Wetzlar, Germany).

### 4.8. Transwell Assay to Determine the Efficacy of Cell Migration

Typically, 1 × 10^5^ cells were cultured in upper transwell chambers in 200 μL of serum-free medium and exposed to different medication regimens. In the lower chambers, 600 μL of medium containing 10% FBS was added. After 48 h of incubation, the cells that did not migrate, located on the upper surface of the chamber, were gently removed using a cotton swab. The migrated cells were then fixed with 4% paraformaldehyde and stained with 0.2% crystal violet.

### 4.9. Detection of SOD/MDA and Iron Using a Specific Testing Kit

The intracellular levels of SOD, MDA, and iron were quantified using specific detection kits (Abcam, Cambridge, MA, USA) according to the manufacturer’s instructions. After drug treatment, the cells were centrifuged and washed with PBS. They were then lysed thoroughly using a sample preparation solution, and the supernatants were collected by centrifugation at 12,000× *g* for 5 min at 4 °C. SOD activity was measured at 450 nm, MDA content was assessed at 532 nm, and the iron concentration was determined by measuring absorbance at 593 nm.

### 4.10. Western Blotting Analysis for Probing the Target Protein

To detect protein expression of the targeted gene in cell lysate, SDS-PAGE (sodium dodecyl sulfate-polyacrylamide gel electrophoresis) was performed. Following electrophoresis, the proteins were transferred to membranes. The membranes were then incubated overnight at 4 °C with 1:5000 dilutions of the following primary antibodies: anti-NRF2 (CST, Danvers, MA, USA), anti-SLC7A11 (CST), anti-GPX4 (CST), anti-Nrf2 (CST), and anti-HO-1 (CST). The anti-tubulin antibody (CST) was used at a 1:5,000,000 dilution. The membranes were then incubated with HRP-conjugated secondary antibodies for 3 h at room temperature. After incubation, the immune complexes were visualized using the enhanced chemiluminescence (ECL) method (Amersham Biosciences, Piscataway, NJ, USA). The band intensities were quantified and analyzed using ImageJ software (Version 1.53q).

### 4.11. Laser Confocal Microscope to Verify the Localization of Target Protein and DAPI

To observe NRF2 translocation, an Olympus Fluoview FV1000 confocal system, equipped with a 63× objective and mounted on an inverted Olympus microscope (Evident Corporation, Tokyo, Japan), was used. The cells were fixed with 4% methanol-free paraformaldehyde for 10 min and then incubated overnight at 4 °C with the primary antibody, diluted in cold PBS containing 2.5% fetal bovine serum and 0.1% Triton X-100 (Sigma-Aldrich). The samples were incubated with the primary antibody in a cold room. For secondary antibody detection, a 1:1000 dilution of FITC-labeled anti-rabbit antibody from Jackson Laboratories (West Grove, PA, USA) was applied. The secondary antibody was incubated for three hours at room temperature in the dark. Following incubation, the cells were washed three times, and the nucleus was stained with DAPI (1:5000 dilution) prior to analysis.

### 4.12. Animal Experiment

Balb/c nude mice (8–10 weeks old) were purchased from Wuhan Shengyu Company in Wuhan, China. The mice were kept in a pathogen-free facility, with a temperature maintained at 22 ± 2 °C, a relative humidity of 70%, and a 12 h light/dark cycle. A549 cells were injected subcutaneously into the flanks of the mice. Tumors became palpable and measurable six days post-injection. The mice were then randomly assigned to one of five treatment groups: the vehicle control group received Milli-Q water, the PTX group received PTX at a dose of 2 mg/kg, the DBT group received the ancient herbal formula at a dose of 10 mg/kg, the DBT + PTX group received 10 mg/kg DBT and 2 mg/kg PTX, and the DBT + PTX + Fer-1 group received 10 mg/kg DBT, 2 mg/kg PTX, and 2 mg/kg Fer-1. To minimize bias, tumor volume measurements and histopathological scoring were performed by investigators who were blinded to the treatment group assignments. Each group has 6 animals for the in vivo experiments. All experimental procedures were approved by Zunyi Medical University’s Animal Experimentation Ethics Committee (Approval No. 21-015) and followed the “Principles of Laboratory Animal Care”. The body weight of the animals was measured every three days, and after 21 days of therapy, the mice were euthanized with Ketamine 100 mg/kg/i.p. and xylazine 10 mg/kg/i.p. Tumor weights and volumes were recorded.

### 4.13. Bioluminescence

Following isoflurane anesthesia, the mice were intraperitoneally injected with 100 μL of aminoluciferin solution (0.5 mM) diluted in saline. After a 3 min incubation period, the mice were euthanized for further analysis. Bioluminescence imaging was performed using the PerkinElmer IVIS^®^ Spectrum imaging system in Alameda, CA. Photographs were taken to capture the bioluminescent signals emitted by the aminoluciferin-labeled cells or tissues using the imaging system.

### 4.14. IHC and HE Staining

The sections were subjected to immunostaining using primary antibodies against Ki67 (CST) and PCNA (CST) at a dilution of 1:200. The sections were incubated with the primary antibodies overnight. Afterward, secondary antibodies at a dilution of 1:500 were applied and incubated for 3 h. The stained sections were examined and photographed using a Nikon light microscope (Nikon, Tokyo, Japan) at a magnification of 200×. Histological changes in the tumors, hearts, kidneys, and livers were evaluated using the H&E staining kit from Abcam. This kit enables the visualization of structural details and cellular morphology in the tissue sections.

### 4.15. TEM Assay

The tumor tissues were prepared for transmission electron microscopy (TEM) imaging by cutting them into thin sections (less than 100 nm thick) using an ultramicrotome. These sections were then stained with lead citrate and 2% uranyl acetate. The staining helps enhance the contrast of the cellular structures under the electron beam. Finally, the stained sections were imaged using a Philips CM100 transmission electron microscope (Philips, Amsterdam, The Netherlands). This microscope allows for high-resolution imaging of the ultrastructural details of the tumor tissues at the cellular level.

### 4.16. Biochemical Analysis

The serum levels of alanine aminotransferase (ALT), aspartate aminotransferase (AST), and alkaline phosphatase (ALP) were determined using detection kits from Abcam. The manufacturer’s instructions for each kit were followed to perform the assays. These kits likely include reagents and protocols specific for the detection and quantification of ALT, AST, and ALP in the serum samples. By following the instructions provided by Abcam, the enzyme activity levels of ALT, AST, and ALP in the serum can be determined accurately.

### 4.17. Data Analysis and Protein Assays

Protein concentrations were determined using BSA (bovine serum albumin) and the protein assay dye from Bio-Rad Laboratories (Hercules, CA, USA). The Bio-Rad Bradford protein assay dye is widely used for quantifying protein concentrations in samples. All data are expressed as mean ± SEM. Normality was assessed using the Shapiro–Wilk test, and homogeneity of variance was assessed using Levene’s test. For multiple comparisons, a one-way ANOVA followed by Bonferroni’s post hoc test was conducted. This post-test was used to identify significant differences between groups when comparing multiple groups simultaneously.

## 5. Conclusions

The combination of PTX and DBT effectively inhibits NSCLC tumor growth by inducing ferroptosis, as demonstrated by the increased levels of lipid peroxidation following treatment with PTX and DBT. By targeting the ferroptosis-driven disruption of the Nrf2/GPX4 axis, DBT enhances PTX’s therapeutic efficacy, potentially overcoming drug resistance and reducing toxicity to healthy cells. Notably, this combination therapy exhibits a favorable safety profile, making it a promising candidate for further clinical exploration. These findings advance our understanding of the anti-cancer mechanisms behind the PTX + DBT combination and highlight its potential as an effective treatment strategy for NSCLC. Further research and clinical trials are needed to confirm these results and refine the dosage and treatment protocols for DBT in combination with PTX. Overall, this study offers valuable insights into the potential of TCM as a complementary approach to boost the efficacy of chemotherapy drugs in NSCLC treatment.

## Data Availability

The data used and/or analyzed during the current study are available from the corresponding author on reasonable request.
